# *Securin* (*hPTTG1*) expression is regulated by *β*-catenin/TCF in human colorectal carcinoma

**DOI:** 10.1038/sj.bjc.6603155

**Published:** 2006-05-16

**Authors:** F Hlubek, S Pfeiffer, J Budczies, S Spaderna, A Jung, T Kirchner, T Brabletz

**Affiliations:** 1 Department of Pathology, Ludwig-Maximilians University of Munich, Thalkirchner Str. 36, 80337 Munich, Germany; 2 oligene GmbH, Campus Charité Mitte, Schumannstr. 20/21, 10117 Berlin, Germany; 3 Department of Pathology, University of Erlangen-Nürnberg, Krankenhausstr. 8-10, 91054, Erlangen, Germany

**Keywords:** colorectal carcinoma, *β*-catenin, human pituitary tumour transforming gene, hPTTG1, Wnt-signalling

## Abstract

Overexpression of the transcriptional activator *β*-catenin, mostly owing to loss-of-function mutations of the *adenomatous polyposis coli* (*APC*) tumour suppressor gene, is crucial for the initiation and progression of human colorectal carcinogenesis. Securin is a regulator of chromosome separation and its overexpression has been shown to be involved in different tumour-promoting processes, like transformation, hyperproliferation and angiogenesis, and correlates with tumour cell invasion. However, the molecular mechanism leading to securin overexpression in human colorectal cancer is unknown. Here we show a correlated high expression of *β*-catenin and securin (hPTTG1) in colorectal adenomas and carcinomas and further demonstrate that *securin* is a target of *β*-catenin transcriptional activation. This implies that deregulation of the *β*-catenin/T-cell factor-signalling pathway leads to overexpression of securin in human colorectal cancer, which subsequently may contribute to tumour progression.

Loss-of-function mutations in the *adenomatosis polyposis coli* (*APC*) tumour suppressor gene, resulting in deregulation of the WNT-signalling pathway, has been associated with the progression of the majority of colorectal carcinomas. The effect is an accumulation of the coactivator protein *β*-catenin, which translocates to the nucleus and functions as a composite transcription factor together with the DNA-binding proteins of the T-cell factor (TCF/LEF) family (Korinek *et al*, 1997). Several genes important for development and tumorigenesis have been reported to be regulated by *β*-catenin/TCF (see for review Brabletz *et al*, 2002).

The human *securin* (*human pituitary tumour transforming gene, hPTTG1*) is an oncogene with little expression in normal adult colon tissue (Zhang *et al*, 1999) in contrast to strong expression in colorectal cancer (Heaney *et al*, 2000) and other human tumours (Dominguez *et al*, 1998; Kakar, 1998; Shibata *et al*, 2002) and several carcinoma cell lines (Kakar, 1998; Zhang *et al*, 1999). Securin is essential for sister chromatid separation during mitosis thereby regulating cell proliferation and chromosome stability (Zou *et al*, 1999). Loss of securin function increases aneuploidy (Jallepalli *et al*, 2001; Wang *et al*, 2001) and apoptosis, in part by modulating p53 function (Yu *et al*, 2000; Bernal *et al*, 2002; Hamid and Kakar, 2004). When overexpressed, securin increases *c-myc* expression and results in elevated cell proliferation and non-tumour cell transformation (Zhang *et al*, 1999; Hamid and Kakar, 2004). Furthermore, high expression of securin has been shown to induce angiogenesis, possibly by activating basic fibroblast growth factor and vascular endothelial growth factor expression (Zhang *et al*, 1999; Ishikawa *et al*, 2001), and to correlate with tumour cell invasion and metastasis (Zhang *et al*, 1999; Heaney *et al*, 2000; Ramaswamy *et al*, 2003). Therefore, securin may contribute to three important ‘hallmarks of cancer’ (Hanahan and Weinberg, 2000): transformation (‘self-sufficient growth’), angiogenesis and invasion.

The molecular mechanism of *securin* regulation in colorectal cancer is unknown. We investigated whether deregulation of the *β*-catenin/TCF-signalling pathway leading to the accumulation of *β*-catenin in colorectal carcinoma causes high expression of *securin* in these tumours, which may contribute to tumour progression and poor prognosis.

## MATERIALS AND METHODS

### Tissue specimen and immunohistochemistry

Formalin-fixed, paraffin-embedded colorectal adenocarcinomas from patients who underwent surgery without additional treatments were retrieved from the archive of the Department of Pathology, University of Erlangen-Nürnberg. The study comprised 30 cases. Immunohistochemistry for *β*-catenin and Ki-67 was performed as described previously (Hlubek *et al*, 2004). For securin staining, a mouse anti-securin antibody (1 : 25; NovoCastra NCL-SECURIN clone DCS-280.2, Newcastle, UK) and microwave treatment for antigen retrieval was used.

### RNA isolation and DNA microarray analysis

Detailed protocols and results will be published elsewhere (Hlubek *et al*, in preparation). This study comprised well characterised moderate-to-well differentiated colorectal adenocarcinomas and mucosa from six patients. Total RNA was extracted from each tissue sample using the RNeasy kit (Qiagen, Hilden, Germany) including an DNA digest according to the manufacturer's instructions. Linear amplification of RNA was carried out with the MessageAmp aRNA kit (Ambion, Huntingdon, UK). Biotin-labelled cRNA was hybridised to the HG-U133A oligonucleotide microarray (22283 probe sets, Affymetrix, Santa Clara, CA, USA). All reactions were performed according to the Affymetrix protocol.

For statistical analysis, the signals of each probe set were summarised to a single expression value according to the standard protocol of the chip manufacturer (MAS 5.0). The expression values were transformed to log-2 scale and normalised by a non-linear procedure based on a scatterplot smoother (Cleveland, 1977). All samples under investigation were normalised against one expression signature that was used as reference. We made use of Welch's two-sample *t*-test in order to detect differential expression between tumour tissue (T) and normal mucosa (N). *P*-values were calculated and corrected in the multiple testing context in order to obtain control over the false discovery rate (FDR), that is, the fraction of false positives in a candidate list of probe sets. Making use of the Benjamini–Hochberg procedure (Benjamini and Hochberg, 1995) we identified a list of 3018 candidates from a total number of 22 283 probe sets (set FDR=5%).

Special attention was paid to targets of the Wnt-signalling pathway. A list of known *β*-catenin/TCF target genes was obtained from the homepage of Joel Nusse (http://www.stanford.edu/~rnusse/wntwindow.html) and expanded by recent reports found in the literature (see for review Brabletz *et al*, 2005). Our list contained a total number of 45 *β*-catenin/TCF target genes of which 42 were represented by one or more probe sets on the GeneChip array. We identified a cluster of 13 target genes (including *securin*) that are (i) significantly regulated in the sense that they are represented by at least one probe set contained in the list of 3018 candidates and (ii) upregulated in tumour tissue compared to normal mucosa.

### Electromobility shift assay

The following sense and corresponding antisense oligonucleotides were annealed and used as probes or for competition (500- and 100-fold molar excess): securin-TBE: 5′-ATAAATCACTATCAAAGGATAGAATTT-3′; securin-mutTBE: 5′-ATAAATCA CT**GC**CAAAGGAT AGAATTT-3′. Probes were end labelled and incubated with 0.5 *μ*g of bacterially expressed GST-TCF-4 DBD (DNA-binding domain codon 265–496) or GST alone as described previously (Hlubek *et al*, 2004).

### DNA clones

The *securin* promoter–reporter plasmid (pGL-Sec. fl.) was constructed by amplification of the primers U1 and D1 described previously (Kakar, 1999) using the bacterial artificial chromosome clone #RP11-35508 (BioCat, Heidelberg, Germany) as template. The PCR product (nt −1336 to +34; GenBank accession number AF167560) was cloned into the pGL3basic (Promega, Mannheim, Germany) reporter vector. For construction of the securin promoter fragment (nt −1336 to 625; pGL-Sec frag.), the primers U1 and PTTG1-625 (5′-TTAAAAAATAAATCGAGAGGCTTT-3′) were used. Plasmids provided by other reseachers are as follows: pGST-TCF4(DBD), pcDNA/hTCF4 and pcDNA/DN-TCF4 from Bert Vogelstein (Johns Hopkins University, Baltimore, MD, USA); pcDNAh*β*-catenin from Hans Clevers (University Medical Center, Utrecht, The Netherlands).

### Transfections and reporter assays

Human colon carcinoma cell lines SW480, DLD1 and HeLa (human cervix carcinoma) cells were obtained from the American Type Culture Collection (ATCC). Cells were cultivated under standard conditions in Dulbecco's modified Eagle's medium +10% fetal bovine serum. Transfections and reporter assays were carried out as described previously (Hlubek *et al*, 2004). Experiments were conducted in triplicate and repeated at least three times with similar results. Control plasmid (pcDNA3; Invitrogen, Karlsruhe, Germany) was used to equalise for the total amount of DNA and to keep the amount of background plasmid constant.

### RNA interference and quantitative real-time reverse-transcription–PCR

Synthetic siRNA (Thermo Electron, Ulm, Germany) specific for *β*-catenin (CAGUUGU GGUUAAGCUCUUdTdT) or GFP (AAGCUACCUGUUCCAUGGCCAdTdT) as control and Oligofectamine (Invitrogen, Karlsruhe, Germany) was used for transient transfection of the colorectal cancer cells according to the instructions of the manufacturer. After 72 h incubation time, cells were harvested, total RNA isolated and real-time reverse transcription—PCR (RT–PCR) was performed as described (Hlubek *et al*, 2004). The relative quantities of securin-specific mRNA in *β*-catenin-siRNA- and control-siRNA-transfected cells were determined for each sample based on the *C*_t_ value from a standard curve generated for each primer/probe set and normalised to the corresponding values of the house-keeping gene *β-actin*.

## RESULTS

### Overexpression of *securin* correlates with nuclear *β*-catenin and Ki-67 expression in human colorectal adenoma and carcinoma

**Figure 1:**
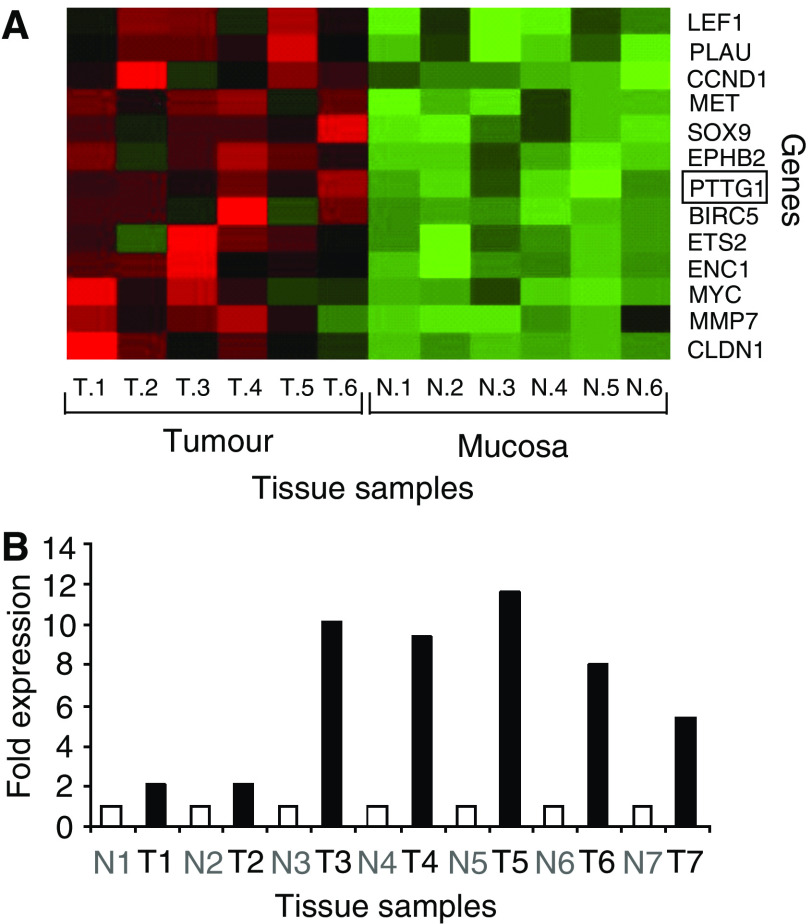
Similar expression pattern of *securin (hPTTG1)* and 12 established *β*-catenin/TCF target genes in tumour *vs* mucosa tissue revealed by hierarchical clustering of microarray data (**A**). All 13 genes are significantly upregulated in colorectal tumour tissue (T.1–T.6) compared to corresponding normal mucosa (N.1–N.6). Gene expression data are displayed as a heatplot with high expression encoded in red and low gene expression shown in green relative to the mean expression (black). Columns represent tissue samples with the corresponding expression level of the individual genes located in rows. Overexpression of *securin* in colorectal carcinoma (T) compared to mucosa (N) of seven different cases analysed by real-time RT–PCR (**B**). *Securin*-specific mRNA expression in seven tumour tissues relative to corresponding normal mucosa was determined following normalisation to the matching expression values of the housekeeping gene *β*-*actin*.

**Figure 2:**
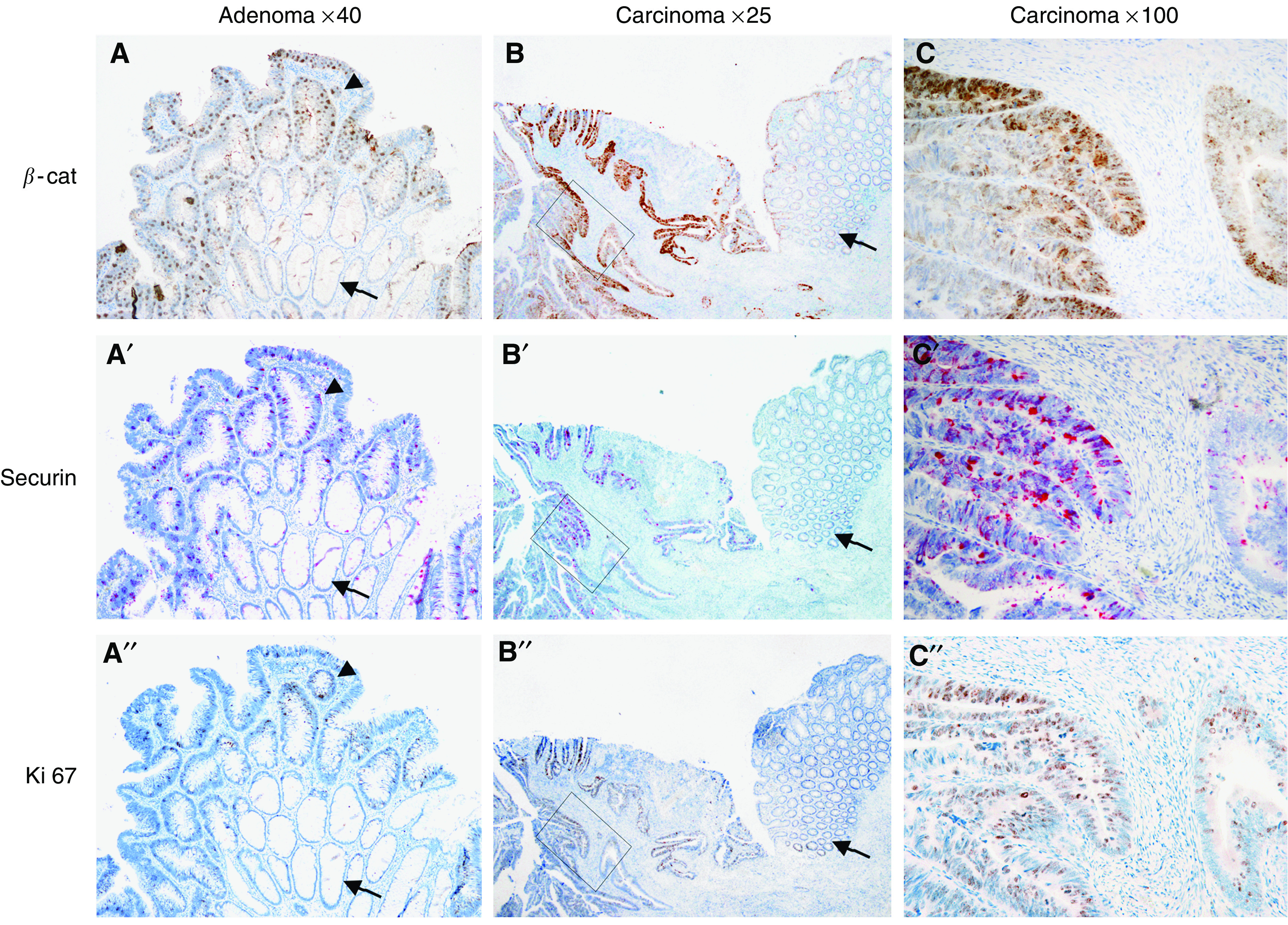
Correlated overexpression of nuclear *β*-catenin, securin and Ki-67 in colorectal adenoma and carcinoma. Immunohistochemical detection of *β*-catenin (first row), securin (second row) and Ki-67 (third row) using serial sections. Tissue sections show adenoma (a, a′, a′′), carcinoma (c, c′, c′′) and the normal mucosa (arrows in a, a′, a″ and b, b′, b′′) of the same case. Note that securin is already overexpressed in the adenoma tissue (arrowhead in a′) and only weakly expressed in the underlying normal colon mucosa (arrow in a′). A substantial overexpression of securin, Ki-67 and nuclear *β*-catenin can be seen in the carcinoma part (b, b′, b′′ and c, c′, c′′; the rectangle in b indicates the magnified area shown in c). Concordant with securin function in mitosis, the proliferation marker Ki-67 displayed the same expression pattern as securin. Nuclear accumulation of *β*-catenin in many of these tumour cells may suggest a function of this transcriptional activator protein for *securin* expression. Magnifications are indicated at the top of the figure.

DNA microarray analysis revealed significant overexpression of *securin* (threefold, *P*-value <0.00001) in six human colorectal carcinomas compared to the corresponding normal mucosa (Figure 1A; detailed results will be published elsewhere (Hlubek *et al*, in preparation). Similarly, *securin* overexpression was found by real-time PCR analysis of seven different colorectal tumours compared to the corresponding mucosa (Figure 1B). Thus, *securin* shows a very similar differential expression pattern between tumour and mucosa tissue like 12 other well-characterised *β*-catenin/TCF target genes (fold changes between 3.3 and 1.8-fold, *P*-value <0.005; Figure 1A). This observation gave rise to the hypothesis, that *securin* is a target gene of *β*-catenin/TCF signalling, which is constitutively active in these tumours. Therefore, we analysed the expression pattern of *β*-catenin and securin protein in 30 colorectal adenocarcinomas by immunohistochemistry. We found a perfect correlation of aberrant nuclear *β*-catenin expression and securin overexpression in colorectal carcinoma and adenoma tissue, in contrast to very low and restricted expression of securin at the colon crypt base in normal mucosa. In line with securin function in mitosis, the proliferation marker Ki-67 displayed the same expression pattern as securin and nuclear *β*-catenin, indicating high proliferative activity of these tumour cells (Figure 2 and Supplementary Figure 1). These results support the hypothesis that accumulated *β*-catenin is an activator of *securin* expression in human colorectal carcinoma.

### *β*-Catenin binds to a TCF-binding site of the human *securin* promoter

**Figure 3:**
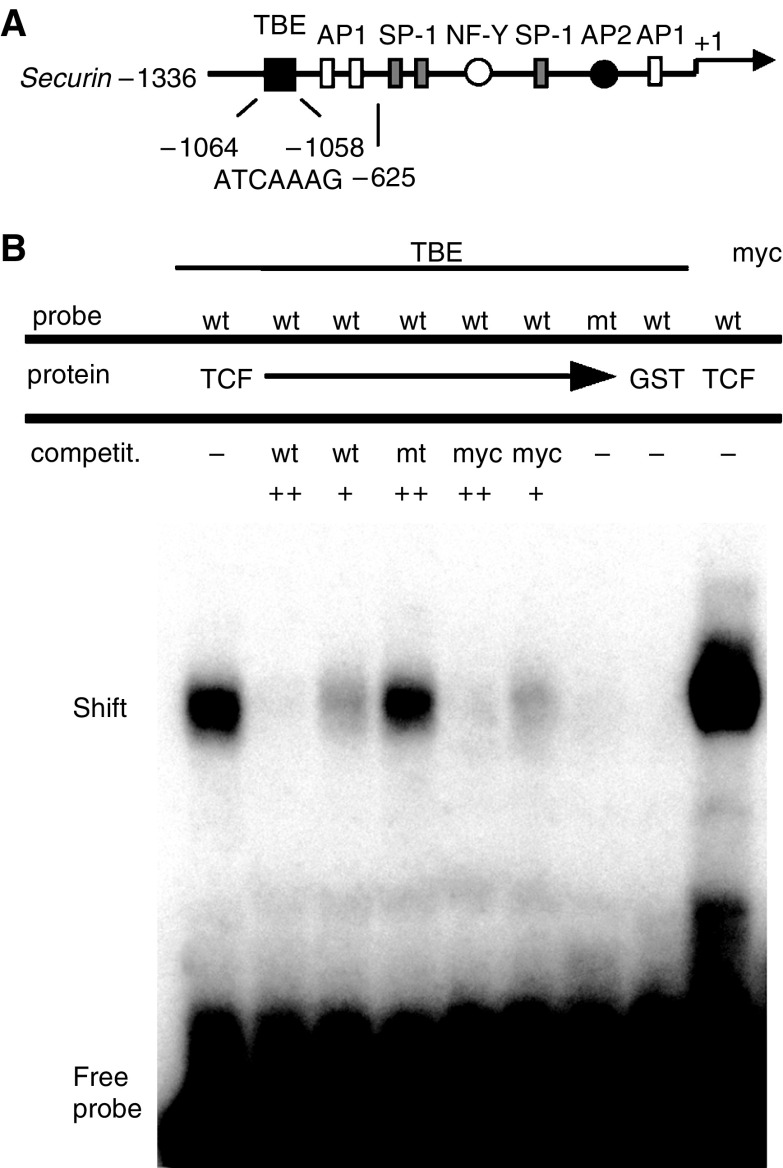
Identification of a TCF-binding element in the human *securin* promoter. Structure of the human *securin* promoter (**A**). The TBE motif is shown as a closed box and the corresponding nucleotide sequence as well as the location relative to the transcription initiation site is indicated below (not drawn to scale). Known transcription factor binding sites are shown (Kakar, 1999; Clem *et al*, 2003). Electromobility shift assay shows specific interaction of recombinant TCF-4 DNA-binding domain (TCF) with oligonucleotides containing the wild-type TBE motif (TBE, wt) of the *securin* promoter by complex formation (**B**, shift). Oligonucleotides were used as labelled probes and as unlabelled competitors (competit.) in two different amounts (wt, wild type; mt, mutant; myc, TBEs of the *c-myc* promoter as positive control). Competition of wild-type *securin* TBE and *c-myc* TBE, but not mutant TBE, indicates specific TCF-4 protein binding. The wild-type DNA probe does not bind to GST protein alone nor does TCF bind to mutant TBE (**B**).

We searched for potential TCF-binding elements (TBE) in the *securin* promoter/enhancer sequence (Kakar, 1999; Clem *et al*, 2003) as *β*-catenin/TCF might directly regulate *securin* expression. Two sites comprising the TBE motif (5′-WWCAAAG-3′) were identified (nt −1064 to −1058, nt −796 to −790) and tested for interaction with TCF-4 in electromobility shift assays (EMSA). One TBE (nt −1064 to −1058; Figure 3A) was found to bind recombinant TCF-4 (Figure 3B), which is consistent with the very recent report by Zhou *et al* (2005). The TCF–DNA interaction was abrogated by specific competitor DNA containing the wild-type TBE site (wt) whereas the mutant TBE motif competitor (mt) had no effect (Figure 3B). Competitor DNA containing the TBE of the c-myc promoter (myc) inhibited TCF binding to TBE to the same extent. Mutant TBE-DNA showed no TCF binding and GST protein alone did not bind to wild-type TBE (Figure 3B), indicating specific binding of TCF-4 to TBE of the *securin* promoter *in vitro*.

### The human *securin* promoter is activated by *β*-catenin/TCF

**Figure 4:**
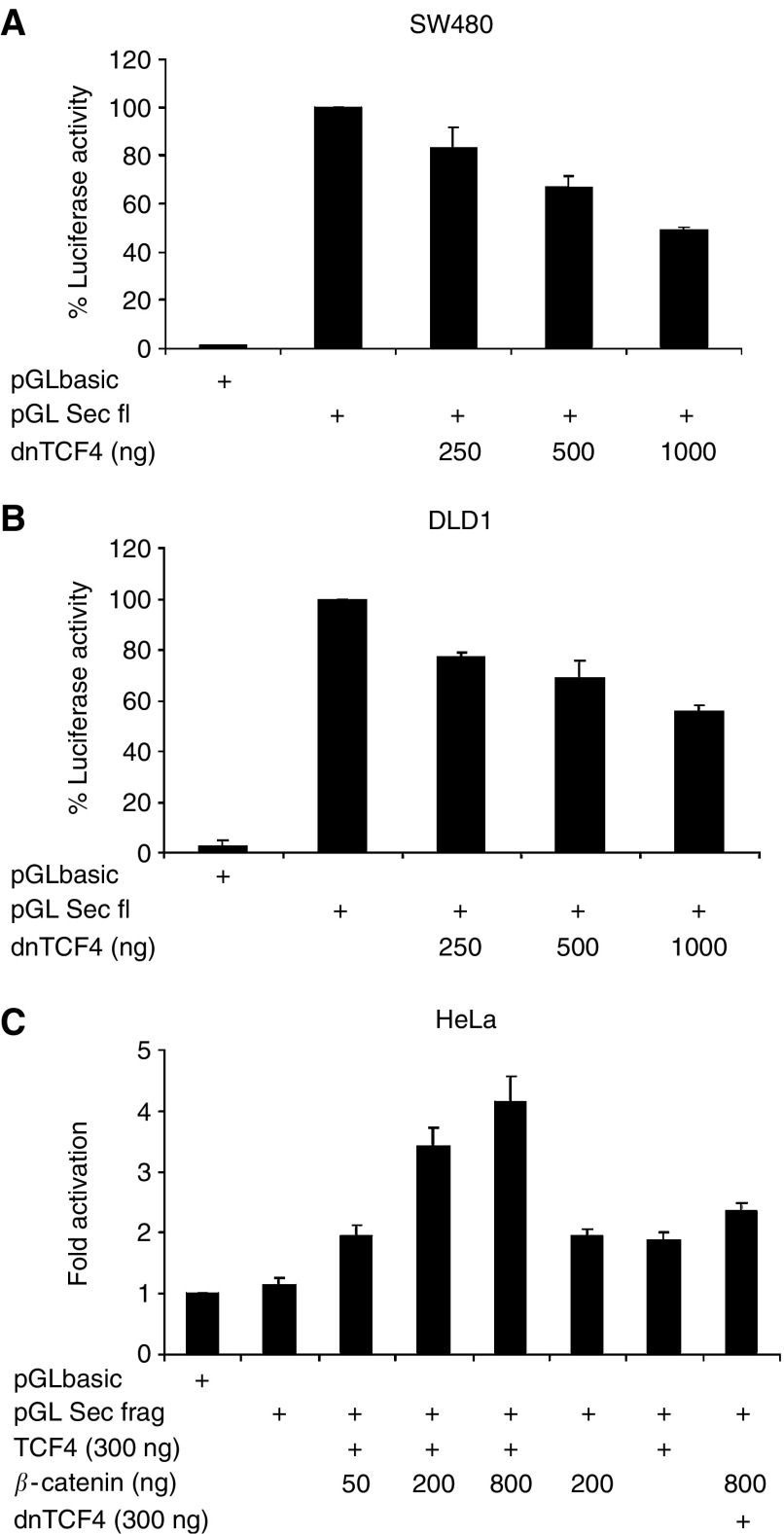
The *β*-catenin/TCF complex activates the human *securin* promoter activity. Inhibition of *securin* promoter activity by dnTCF in colorectal carcinoma cells with constitutively active *β*-catenin. The results of the transient transfection reporter assays are shown as percentage of full-length *securin* promoter activity (pGL Sec fl.; pGLbasic, vector control; **A, B**). *Securin* promoter fragment (pGL Sec frag.) activity is enhanced by *β*-catenin and TCF-4 in a dose-dependent manner. In contrast, transfection of either *β*-catenin or TCF-4 alone does not augment *securin* promoter activity (**C**). Results are represented as fold activation of promoter activity by transfection of increasing amounts of *β*-catenin related to the vector control transfected (pGLbasic). Cotransfection of dnTCF inhibits the augmentation of *securin* promoter fragment activity induced by *β*-catenin. The experiments were carried out in triplicate and repeated three times with similar results.

The functional relevance of TCF-4 binding to the TBE motive was investigated in luciferase reporter assays using a full-length securin promoter (−1336 to +34). In *β*-catenin-overexpressing colorectal carcinoma cell lines SW480 and DLD1, the *securin* promoter (pGL Sec fl.) exhibits strong transcriptional activity, 74 and 35-fold over background (pGLbasic). Addition of dominant-negative TCF-4 (dnTCF), which lacks the N-terminal *β*-catenin interaction domain, reduced the *securin* promoter activity in a dose-dependent manner by up to 51 and 44 % (Figure 4A and B). As it is known that complete abrogation of *β*-catenin/TCF transcriptional activity by dnTCF can induce a G1-phase arrest of the cell cycle (van de Wetering *et al*, 2002) possibly influencing the cell cycle-dependent expression of securin, we determined cell cycle progression by fluorescence-activated cell sorting (FACS) analysis (Supplementary Figure 2). Unsynchronised DLD1 and SW480 cells showed no significant change in cell cycle phases after transient transfection with dnTCF compared to cells transfected with control vector.

Furthermore, in HeLa cells, which do not have an activated *β*-catenin/TCF-signalling pathway, the activity of the *securin* promoter fragment (−1336 to −625) was enhanced by overexpression of TCF4 and *β*-catenin in a dose-dependent manner (Figure 4C). Neither *β*-catenin nor TCF alone was able to activate the *securin* promoter fragment. In addition, dnTCF abolished promoter activation by *β*-catenin (Figure 4C).

### Expression of endogenous human *securin* is regulated by *β*-catenin

**Figure 5:**
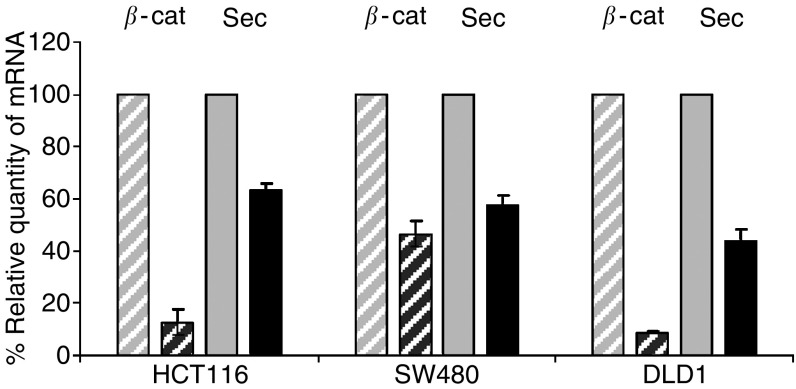
Endogenous *securin* expression is activated by *β*-catenin. HCT116, SW480 and DLD1 colorectal cancer cells were transfected with *β*-catenin-specific siRNA and control siRNA (GFP-siRNA). The effect of *β*-catenin knockdown on *securin* expression was measured by quantitative real-time RT–PCR and *β*-catenin expression was confirmed by Western blotting (Supplemental Figure 3). Inhibition of *β*-catenin expression by siRNA in colorectal cancer cells clearly reduces the endogenous *securin* expression. Black bars: *β*-catenin siRNA-transfected cells; grey bars: control cells (GFP-siRNA transfected); hatched bars: quantity of *β*-catenin mRNA; closed bars: quantity of securin mRNA. Quantification of securin and *β*-catenin-specific mRNA was done in triplicates, the amounts were normalised to the amount of *β*-actin mRNA, and expressed in percent relative to the control cells. The experiments were repeated twice with similar results.

To test the regulatory function of *β*-catenin on the expression of the endogenous *securin* gene in colorectal carcinoma cells, we utilised siRNA knockdown technology. The expression of endogenous *β*-catenin was inhibited by transfection of HCT116, SW480 and DLD1 colon cancer cells with *β*-catenin-specific siRNA. *β*-catenin expression, measured by quantitative RT–PCR, was clearly reduced compared to the control cells (transfected with green fluorescent protein (GFP)-siRNA; Figure 5). Specific reduction of *β*-catenin expression by *β*-catenin-specific siRNA transfection was confirmed by Western blot in control experiments (Supplementary Figure 3; see also Hlubek *et al*, 2004). Importantly, transfection of *β*-catenin-specific siRNA decreased endogenous *securin* expression by 37, 43 and 56% compared to control cells, but not *β-actin* expression (Figure 5, and Supplementary Figure 3). To exclude the possibility that reduction of *securin* expression was caused by changes in cell cycle progression, we performed control experiments using FACS analysis. Transient transfection of HCT116 cells with *β*-catenin-specific siRNA did not result in significant changes in the cell cycle phase distribution (Supplementary Figure 4).

## DISCUSSION

The results presented above demonstrate a correlated expression of *β*-catenin, securin and Ki-67 in human colorectal adenomas and carcinomas, indicating that *securin* is a potential *β*-catenin/TCF target gene. In support of this hypothesis, we show that *securin*, like 12 well-known *β*-catenin/TCF target genes (e.g. *c-myc*, *c-Met*, *survivin* and *MMP7*), is significantly overexpressed in colorectal cancer compared to normal mucosa using DNA microarray analysis, real-time PCR and immunohistochemistry. Moreover, we found a functional TCF-binding element in the *securin* promoter sequence identical to the TBE found to be functional in esophageal squamous cell carcinoma, published during the preparation of this manuscript (Zhou *et al*, 2005). In EMSA experiments, we show specific TCF–DNA interaction. The functional relevance of this interaction is shown by reporter-gene experiments in colorectal cancer cell lines demonstrating that dnTCF inhibits *securin* promoter activity in a dose-dependent manner. As *securin* expression is cell cycle dependent, peaking in mitosis (Ramos-Morales *et al*, 2000), and *β*-catenin can influence cell cycle progression by regulating *c-myc* expression (van de Wetering *et al*, 2002), we performed FACS anaylsis. However, we did not observe significant differences in cell cycle progression beween dnTCF-transfected and control vector-tranfected cells (Supplementary Figure 2). The transient transfection of dnTCF or *β*-catenin siRNA (see below) reduces *β*-catenin transcriptional activity, but does not completely abolish it, which would be necessary for inducing a G1-phase arrest of the cell cycle (van de Wetering *et al*, 2002, 2003).

Additionally, we show that *securin* promoter fragment containing the TBE is activated in HeLa cells by overexpression of TCF-4 and *β*-catenin, but not by either protein alone. However, we were unable to activate the full-length promoter construct significantly by overexpression of TCF-4 and *β*-catenin, because the promoter region (659 nucleotides) proximal to the transcriptional start site contains additional regulatory elements (Kakar, 1999; Clem *et al*, 2003) presumably obscuring this effect. We tested the function of *β*-catenin/TCF in the regulation of the endogenous *securin* gene by transfection of colorectal carcinoma cells with *β*-catenin-specific siRNA. Owing to the transient transfection procedure used, no complete abrogation of *β*-catenin expression was observed and cell cycle progression was not changed. However, a potent inhibition of *β*-catenin expression was induced, resulting in a significant reduction of the endogenous *securin* gene expression. Even though we do not exclude the possibility of an indirect effect of *β*-catenin on *securin* expression by affecting cell cycle progression, our results show the relevance of *β*-catenin for the direct regulation of *securin* gene expression in colorectal tumour cells.

The oncogene *securin* is deregulated in many human tumours and is able to contribute to several main aspects of tumorigenesis. In several tumours, overexpression of securin is associated with tumour metastasis and poor clinical outcome and has been identified as a significant prognostic marker in esophageal carcinoma (Zhang *et al*, 1999; Heaney *et al*, 2000; Shibata *et al*, 2002; Ramaswamy *et al*, 2003). The identification of *securin* as a Wnt target gene activated early in the adenoma–carcinoma sequence further supports a decisive role of aberrant overexpression of *β*-catenin in all phases of colorectal carcinogenesis.

## References

[bib1] Benjamini Y, Hochberg Y (1995) Controlling the false discovery rate: a practical and powerful approach to multiple testing. J Royal Stat Soc Ser B 57: 289–300

[bib2] Bernal JA, Luna R, Espina A, Lazaro I, Ramos-Morales F, Romero F, Arias C, Silva A, Tortolero M, Pintor-Toro JA (2002) Human securin interacts with p53 and modulates p53-mediated transcriptional activity and apoptosis. Nat Genet 32: 306–311 12355087 10.1038/ng997

[bib3] Brabletz T, Hlubek F, Spaderna S, Schmalhofer O, Hiendlmeyer E, Jung A, Kirchner T (2005) Invasion and metastasis in colorectal cancer: epithelial–mesenchymal transition, mesenchymal–epithelial transition, stem cells and b-catenin. Cells Tissues Organs 179: 56–65 15942193 10.1159/000084509

[bib4] Brabletz T, Jung A, Kirchner T (2002) Catenin and the morphogenesis of colorectal cancer. Virchows Arch 441: 1–11 12111194 10.1007/s00428-002-0642-9

[bib5] Clem AL, Hamid T, Kakar SS (2003) Characterization of the role of Sp1 and NF-Y in differential regulation of PTTG/securin expression in tumor cells. Gene 322: 113–121 14644503 10.1016/j.gene.2003.08.012

[bib6] Cleveland W (1977) Robust locally weighted regression and smoothing scatterplots. J Am Stat Assoc 74: 829–836

[bib7] Dominguez A, Ramos-Morales F, Romero F, Rios RM, Dreyfus F, Tortolero M, Pintor-Toro JA (1998) hpttg, a human homologue of rat pttg, is overexpressed in hematopoietic neoplasms. Evidence for a transcriptional activation function of hPTTG. Oncogene 17: 2187–2193 9811450 10.1038/sj.onc.1202140

[bib8] Hamid T, Kakar SS (2004) PTTG/securin activates expression of p53 and modulates its function. Mol Cancer 3: 18 15242522 10.1186/1476-4598-3-18 PMC479695

[bib9] Hanahan D, Weinberg RA (2000) The hallmarks of cancer. Cell 100: 57–70 10647931 10.1016/s0092-8674(00)81683-9

[bib10] Heaney AP, Singson R, McCabe CJ, Nelson V, Nakashima M, Melmed S (2000) Expression of pituitary-tumour transforming gene in colorectal tumours. Lancet 355: 716–719 10703804 10.1016/S0140-6736(99)10238-1

[bib11] Hlubek F, Spaderna S, Jung A, Kirchner T, Brabletz T (2004) Beta-Catenin activates a coordinated expression of the proinvasive factors laminin-5 gamma2 chain and MT1-MMP in colorectal carcinomas. Int J Cancer 108: 321–326 14639622 10.1002/ijc.11522

[bib12] Ishikawa H, Heaney AP, Yu R, Horwitz GA, Melmed S (2001) Human pituitary tumor-transforming gene induces angiogenesis. J Clin Endocrinol Metab 86: 867–874 11158059 10.1210/jcem.86.2.7184

[bib13] Jallepalli PV, Waizenegger IC, Bunz F, Langer S, Speicher MR, Peters JM, Kinzler KW, Vogelstein B, Lengauer C (2001) Securin is required for chromosomal stability in human cells. Cell 105: 445–457 11371342 10.1016/s0092-8674(01)00340-3

[bib14] Kakar SS (1998) Assignment of the human tumor transforming gene TUTR1 to chromosome band 5q35.1 by fluorescence *in situ* hybridization. Cytogenet Cell Genet 83: 93–95 9925941 10.1159/000015139

[bib15] Kakar SS (1999) Molecular cloning, genomic organization, and identification of the promoter for the human pituitary tumor transforming gene (PTTG). Gene 240: 317–324 10580151 10.1016/s0378-1119(99)00446-1

[bib16] Korinek V, Barker N, Morin PJ, van Wichen D, de Weger R, Kinzler KW, Vogelstein B, Clevers H (1997) Constitutive transcriptional activation by a beta-catenin-Tcf complex in APC−/− colon carcinoma [see comments]. Science 275: 1784–1787 9065401 10.1126/science.275.5307.1784

[bib17] Ramaswamy S, Ross KN, Lander ES, Golub TR (2003) A molecular signature of metastasis in primary solid tumors. Nat Genet 33: 49–54 12469122 10.1038/ng1060

[bib18] Ramos-Morales F, Dominguez A, Romero F, Luna R, Multon MC, Pintor-Toro JA, Tortolero M (2000) Cell cycle regulated expression and phosphorylation of hpttg proto-oncogene product. Oncogene 19: 403–409 10656688 10.1038/sj.onc.1203320

[bib19] Shibata Y, Haruki N, Kuwabara Y, Nishiwaki T, Kato J, Shinoda N, Sato A, Kimura M, Koyama H, Toyama T, Ishiguro H, Kudo J, Terashita Y, Konishi S, Fujii Y (2002) Expression of PTTG (pituitary tumor transforming gene) in esophageal cancer. Jpn J Clin Oncol 32: 233–237 12324572 10.1093/jjco/hyf058

[bib20] van de Wetering M, Oving I, Muncan V, Pon Fong MT, Brantjes H, van Leenen D, Holstege FC, Brummelkamp TR, Agami R, Clevers H (2003) Specific inhibition of gene expression using a stably integrated, inducible small-interfering-RNA vector. EMBO Rep 4: 609–615 12776180 10.1038/sj.embor.embor865 PMC1319205

[bib21] van de Wetering M, Sancho E, Verweij C, de Lau W, Oving I, Hurlstone A, van der Horn K, Batlle E, Coudreuse D, Haramis AP, Tjon-Pon-Fong M, Moerer P, van den Born M, Soete G, Pals S, Eilers M, Medema R, Clevers H (2002) The beta-catenin/TCF-4 complex imposes a crypt progenitor phenotype on colorectal cancer cells. Cell 111: 241–250 12408868 10.1016/s0092-8674(02)01014-0

[bib22] Wang Z, Yu R, Melmed S (2001) Mice lacking pituitary tumor transforming gene show testicular and splenic hypoplasia, thymic hyperplasia, thrombocytopenia, aberrant cell cycle progression, and premature centromere division. Mol Endocrinol 15: 1870–1879 11682618 10.1210/mend.15.11.0729

[bib23] Yu R, Heaney AP, Lu W, Chen J, Melmed S (2000) Pituitary tumor transforming gene causes aneuploidy and p53-dependent and p53-independent apoptosis. J Biol Chem 275: 36502–36505 11013229 10.1074/jbc.C000546200

[bib24] Zhang X, Horwitz GA, Prezant TR, Valentini A, Nakashima M, Bronstein MD, Melmed S (1999) Structure, expression, and function of human pituitary tumor-transforming gene (PTTG). Mol Endocrinol 13: 156–166 9892021 10.1210/mend.13.1.0225

[bib25] Zhou C, Liu S, Zhou X, Xue L, Quan L, Lu N, Zhang G, Bai J, Wang Y, Liu Z, Zhan Q, Zhu H, Xu N (2005) Overexpression of human pituitary tumor transforming gene (hPTTG), is regulated by beta-catenin/TCF pathway in human esophageal squamous cell carcinoma. Int J Cancer 113: 891–898 15514942 10.1002/ijc.20642

[bib26] Zou H, McGarry TJ, Bernal T, Kirschner MW (1999) Identification of a vertebrate sister-chromatid separation inhibitor involved in transformation and tumorigenesis. Science 285: 418–422 10411507 10.1126/science.285.5426.418

