# Supplementary figures and images for: Securin (hPTTG1) expression is regulated by β-catenin/TCF in human colorectal carcinoma
Source: Br J Cancer. 2006 May 16;94(11):1672–7. doi: 10.1038/sj.bjc.6603155 (PMC2361298; doi:10.1038/sj.bjc.6603155)

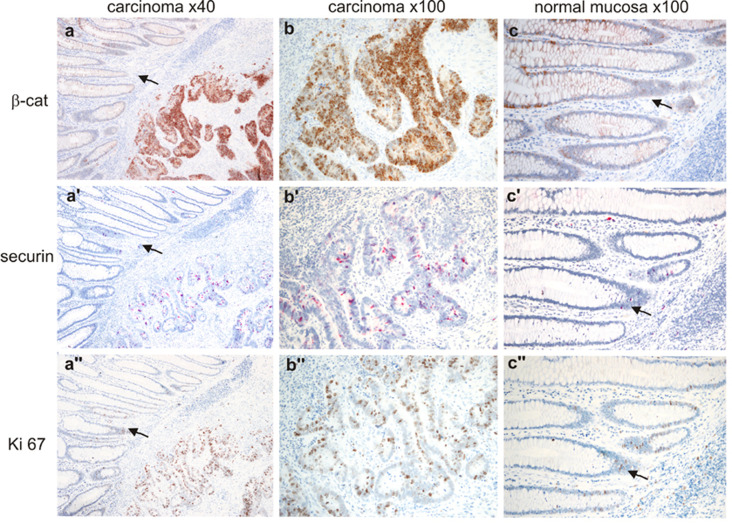

Supplement: Supplemental Figure 1 [file 94-6603155x1.jpg]

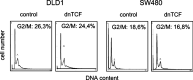

Supplement: Supplemental Figure 2 [file 94-6603155x2.gif]

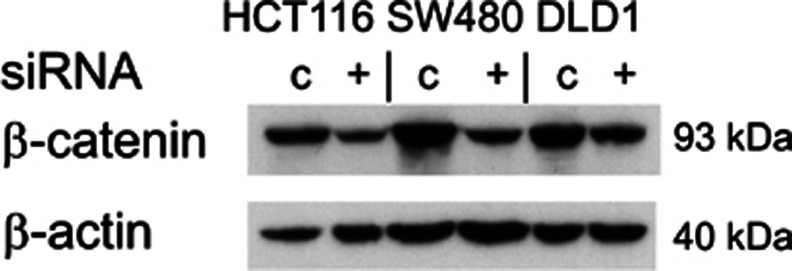

Supplement: Supplemental Figure 3 [file 94-6603155x3.jpg]

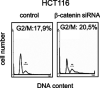

Supplement: Supplemental Figure 4 [file 94-6603155x4.gif]
