# Supplementary material for: Securin (hPTTG1) expression is regulated by β-catenin/TCF in human colorectal carcinoma
Source: Br J Cancer. 2006 May 16;94(11):1672–7. doi: 10.1038/sj.bjc.6603155 (PMC2361298; doi:10.1038/sj.bjc.6603155)
Supplement: Legends to Supplemental Figures [file 94-6603155x5.doc]

**SUPPLEMENTAL FIGURE Legends**

supplemental Fig. 1:

Example of another case with correlated overexpression of nuclear β-catenin, securin and Ki-67 in colorectal carcinoma (b, b', b''). Serial sections were used for immunohistochemical detection of β-catenin (brown; a, b, c), securin (red; a', b', c') and Ki-67 (brown; a'', b'', c''). Again, normal mucosa tissue shows only a rare expression of securin in colon epithelial cells at the crypt base (arrow in c'). Arrows indicate crypt base of normal mucosa tissue.

supplemental Fig. 2:

Transient transfection of dnTCF4 does not affect cell cycle progression. Colorectal carcinoma cell lines DLD1 and SW480 were transfected with the securin-reporter construct and dnTCF4 expression plasmid (dnTCF) or empty control plasmid (control). Identical transfection procedures were used for the experiments shown and the experiments displayed in figure 4. The harvested cells were devided in two portions: One third of the cells was used for the luciferase reporter assay and with the remaining two thirds of the cells FACS analysis was done. Cells transfected with dnTCF4 showed a reduction in securin promoter activity to 73% (DLD1 cells) and to 60% (SW480 cells) compared to control transfectants. This inhibiting effect of dnTCF on the securin promoter activity is similar to the results shown in figure 4. Propidium iodide staining and FACS analysis revealed no significant differences in cell cycle phase distribution between dnTCF4 trans­fected cells and control cells. The percentage of cells in G2/M phase is indicated.

supplemental Fig. 3:

Specific reduction of β-catenin protein expression in colorectal carcinoma cells DLD1, SW480 and HCT116 by transfection of β-catenin-siRNA. In order to prove that β-catenin-siRNA transfection results in specific reduction in β-catenin protein, cells were transfected with β-catenin specific siRNA (+) or control siRNA (GFP-siRNA; c) and harvested after 72 hours incubation time. Cells were divided in two portions: one portion of cells was used for RNA isolation and real-time RT-PCR. The cells of the second portion were lysed and used for protein immunodetection by western blotting. In β-catenin-siRNA transfected cells β-catenin protein expression is reduced compared to the control tranfectants whereas equal amounts of β-actin is detected in both cells. Quantification by real-time PCR resulted in a reduction of β-catenin mRNA to 42,2 % (DLD1), 13,4 % (SW480) and 23,8 % (HCT116) compared to the control cells. Relative quantities were normalized to the corresponding values of the house keeping gene β-actin.

supplemental Fig. 4:

FACS analysis reveals that transient transfection of HCT116 cells with β-catenin-specific siRNA does not result in significant changes in cell cycle progression. Transfected cells were devided in two portions: One portion of cells was used for quantification of β-catenin-specific mRNA by real-time PCR and the second portion was subjected to cell cycle progression analysis by FACS. Propidium iodide staining and FACS analysis reveals no significant differences in cell cycle phase distribution between β-catenin-specific siRNA transfected cells and control cells transfected with GFP-siRNA (control). The percentage of cells in G2/M phase is indicated. Quantification of mRNA indicates a reduction of β-catenin expression level to 13,6 % and securin expression to 67 % compared to the control cells, which is comparable to the results shown in figure 5.
